# Memory effects in annealed hybrid gold nanoparticles/block copolymer bilayers

**DOI:** 10.1186/1556-276X-6-167

**Published:** 2011-02-23

**Authors:** Vanna Torrisi, Francesco Ruffino, Antonino Licciardello, Maria Grazia Grimaldi, Giovanni Marletta

**Affiliations:** 1 Laboratory for Molecular Surfaces and Nanotechnology (LAMSUN), Department of Chemical Sciences, University of Catania and CSGI, Viale A. Doria 6, 95125, Catania, Italy; 2 Dipartimento di Fisica e Astronomia and MATIS CNR-IMM, Università di Catania, Via S. Sofia 64, 95123, Catania, Italy

## Abstract

We report on the use of the self-organization process of sputtered gold nanoparticles on a self-assembled block copolymer film deposited by horizontal precipitation Langmuir-Blodgett (HP-LB) method. The morphology and the phase-separation of a film of poly-*n*-butylacrylate-*block*-polyacrylic acid (P*n*BuA-*b*-PAA) were studied at the nanometric scale by using atomic force microscopy (AFM) and Time of Flight Secondary Ion Mass Spectrometry (TOF-SIMS). The templating capability of the P*n*BuA-*b*-PAA phase-separated film was studied by sputtering gold nanoparticles (NPs), forming a film of nanometric thickness. The effect of the polymer chain mobility onto the organization of gold nanoparticle layer was assessed by heating the obtained hybrid P*n*BuA-*b*-PAA/Au NPs bilayer at *T *>*T*_g_. The nanoparticles' distribution onto the different copolymer domains was found strongly affected by the annealing treatment, showing a peculiar memory effect, which modifies the AFM phase response of the Au NPs layer onto the polar domains, without affecting their surfacial composition. The effect is discussed in terms of the peculiar morphological features induced by enhanced mobility of polymer chains on the Au NPs layer.

## Introduction

Recent advances in the patterning of polymers have enabled the fabrication of integrated micro- and nanosystems with high degree of complexity and functionality. For example, block copolymers have attracted immense interest for nanotechnology applications because of easy processability and low-cost fabrications. The chemically distinct and immiscible polymer blocks in block copolymers microphase-separate and self-assemble into ordered patterns on the scale of nanometers [1-3]. This soft nanostructured polymer film can further be used as a template for patterning of hard inorganic materials such as metal nanoparticles [4-10]. Metal nanoclusters in a matrix of insulating polymer have unique physical properties and have been proposed for optical, electrical and magnetic applications [11-14].

Previous studies demonstrate that metal nanoparticles can preferentially decorate a particular domain in a diblock copolymer film. In general, the specific nature of the selective gold-polymer interaction that causes the self-assembly is still far from being completely understood.

Patterning of metal nanoparticles within polymer films has been achieved using four main routes. The first method is vapour phase co-deposition of polymers/nanoparticles in high vacuum followed by thermal annealing [15-18]. Annealing of the polymer film above the glass transition temperature (*T*_g_) of the polymer allows structural relaxation of the polymer matrix and was proven to be responsible for the dispersion of the metal nanoparticles within the polymer film. The second method is based on the deposition from a mixture of block copolymer and organic-coated nanoparticles in solution onto a solid surface followed by the annealing step [19-25]. The third method employs the dewetting of polymer films made from low concentrations of mixed solutions of polymer and polymer-grafted nanoparticles to create metal nanostructures [26-29]. The fourth method uses the self-organization characteristic of evaporated nanoparticles on a self-assembled polymer film to create nanopatterning by selective adsorption [30].

We used the sputtering technique to investigate the deposition behaviour of gold nanoparticles onto block copolymer template.

## Experimental

### Substrate cleaning and polymer coating

A silicon wafer 100 (p-type, Boron-doped) was cut into 1 × 1 cm^2 ^pieces. The silicon substrates were cleaned as follows: soaking in the cleaning bath at 75°C for 10 min. The cleaning solution was composed of 100 ml of 96% NH_4_OH, 35 ml of 35% H_2_O_2 _and 65 ml deionized water. The cleaned substrates were further rinsed in deionized water for 10 min and finally deposited by horizontal precipitation Langmuir-Blodgett (HP-LB) method [31].

A CHCl_3 _1 mg/ml solution of poly-*n*-butylacrylate-*block*-polyacrylic acid (P*n*BuA-*b*-PAA) (MW 13,000 Da) was used for film deposition by means of HP-LB.

This solution was used for preparation of Langmuir polymer layers at the water/air interface in a computer-controlled trough (LT-102, MicrotestMachines, Belarus). The floating film was compressed at a rate of 0.5 mm/s (or 0.75 cm^2^/s) and the corresponding isotherms were acquired. LB films of each mixture (applied pressure 11-14 mN/m) were transferred on cleaned silicon 100 substrates by means of HP-LB method.

### Gold nanoparticles sputtering deposition

The depositions were carried out using an RF (60 Hz) Emitech K550x Sputter coater apparatus onto the substrates and clamped against the cathode located straight opposite of the Au source (99.999% purity target). The electrodes were laid at a distance of 40 mm under Ar flow keeping a pressure of 0.02 mbar in the chamber. The deposition time was 30 s with working current of 10 mA, corresponding to about 3 nm of deposited Au.

### Annealing treatment

The polymer films were annealed in a vacuum oven at 115°C for 15, 30, 45, 60, 90 min.

### Morphological characterization

AFM images were obtained in tapping mode using a MultiMode Nanoscope IIIa (Digital Instruments, USA). The device is equipped with a *J *scanner, which was calibrated using the manufacturer's grating. Ultrasharp tips (Noncontact "Golden" Silicon cantilevers, NSG10S, typical force constant 11.5 N/m, resonant frequency 255 kHz) were used. Height images are flattened to remove background slopes. No other filtering procedures are performed on these images.

### Chemical imaging

Static SIMS images were acquired with a TOF-SIMS IV (ION-TOF), using a pulsed Bi^+ ^primary ion beam (burst alignment mode, 25 KeV, 0.5 pA, 100 μm × 100 μm raster, PI fluence < 3 × 10^11 ^ions/cm^2^). Detailed images were obtained from small areas (100 μm × 100 μm with 256 × 256 pixel definition) using the high spatial imaging mode. This allows a spatial resolution of about 200 nm; however, mass resolution is greatly degraded. Analyses below the static limit were performed.

## Results and discussion

**Figure 1:**
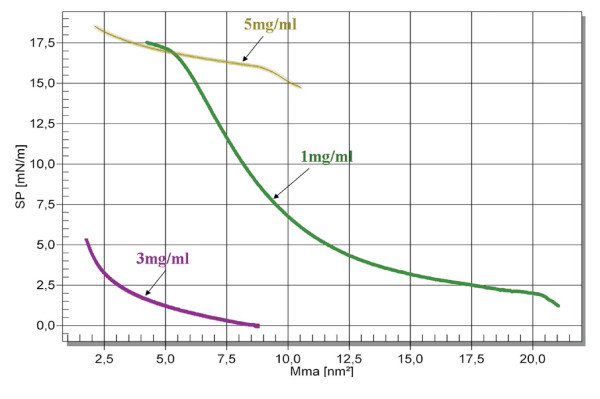
**Surface pressure versus molecular area isotherms obtained by 1, 3, 5 mg/ml chloroform solutions of P*n*BuA-*b*-PAA**.

Figure 1 reports the Langmuir isotherms obtained for P*n*BuA-*b*-PAA films at three different solution concentrations, i.e., 1, 3 and 5 mg/ml. The fact that at a given molecular area (for instance 7,5 nm^2^) the pressure at 3 mg/ml is lower than that one reported in the 1 mg/ml isotherm is unusual. It depends on the characteristic behaviour of block copolymers in Langmuir-Blodgett films and on their pressure-induced reorganization/reorientation phenomena at the air/water interface [32]. The lower surface pressure for the phase transitions of 3 mg/ml with respect to 1 mg/ml isotherm originates from chains reorientations of two blocks. Such reorientations are the result of the balance between block-block and block interface interactions.

In particular, the appearance of a well-defined plateau region around a surface pressure of 17 mN/m for the concentration of 5 mg/ml is diagnostic of the formation of the liquid/solid-like region characteristic of the circular domains. Accordingly, the 5 mg/ml concentration corresponds to the critical micellar concentration (CMC) for the specific P*n*BuA-*b*-PAA employed in this study [33].

Therefore, in order to obtain a well-packed P*n*BuA-*b*-PAA film, the deposition was performed well above the plateau surface pressure, i.e., at a surface pressure of 25 mN/m. According to wide literature, the structure of the film in the solid-like phase region is the result of drastic self-assembling processes of the different polymer blocks, basically yielding circular domains based on PAA chains, protruded towards the water subphase, and a matrix based on P*n*BuA chains, spread at the water/air interface. The transfer of the films onto solid surfaces (silicon) by HP-LB method maintains the lateral inhomogeneity of the film structure [31,34,35].

**Figure 2:**
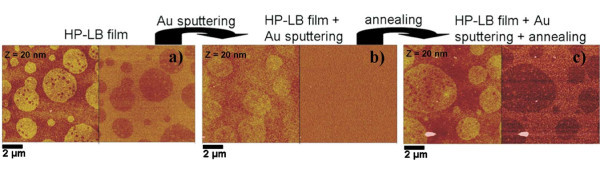
**AFM images of the three steps of sample preparation**: **(a) **HP-LB film of P*n*BuA-*b*-PAA; **(b) **HP-LB film covered with Au nanoparticles deposited by sputtering; **(c) **annealed bilayer (115°C, 15 min).

Atomic force microscopy (AFM) measurements of the films morphology at the microscale are reported in Figure 2a, showing the characteristic formation of higher circular domains, corresponding to the micelles pulled out by the deposition, and a flat matrix, formed by the P*n*BuA blocks. The corresponding phase image, sensitive to the chemical termination of the different regions, clearly shows the different chemical structure of the protruding hydrophilic spots, consisting indeed of PAA blocks, and the flat hydrophobic regions, due to the assembly of P*n*BuA blocks.

Figure 2b shows the effect of the Au sputtering deposition. One can observe the decrease of the height of the hydrophilic PAA-based circular domains with respect to the matrix in the height image, whilst in the phase image, as expected for the homogeneous Au NP coating produced, one can observe a uniform and unstructured image, corresponding to the perfectly homogeneous coating of Au.

By AFM characterization of the annealed bilayer (Figure 2c) we have again evidenced of a phase separation. The nanoparticles' distribution onto the block copolymer domains, studied by AFM, seems strongly affected by the bilayer annealing, showing an apparent return of the initial dephasing of the HP-LB block copolymer film. Regarding the cause of this return of the dephasing we can do some hypothesis: (1) Gold segregation onto the polar domains because of the increased diffusion of gold onto diblock copolymer film during the thermal annealing (higher mobility of gold [36] because of the higher fluidity of polymer chains) and furthermore due to new positioning of gold driven by block copolymer template. (2) The second hypothesis is an in-depth diffusion of gold as Kunz et al. [37] have just observed for discontinuous gold films on amorphous polymer substrates. In fact amorphous polymers behave as viscous fluids at temperatures above glass transition temperatures and such behaviour could induce an increasing of the mobility of gold. (3) Third hypothesis implies a modification of the surface-tip interaction produced by new hardness or viscoelasticity properties of the uppermost layer.

**Figure 3:**
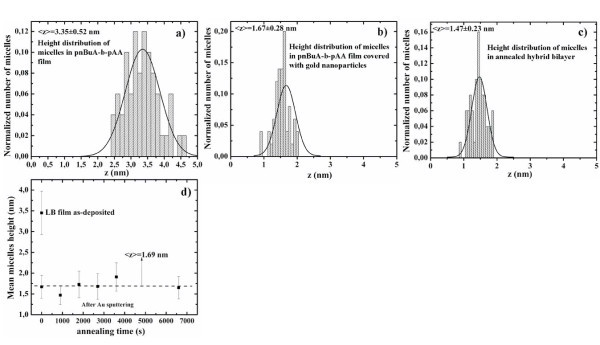
**Height distribution of the micelles after each deposition step of as deposited and annealed samples**: Micelles height versus annealing time **(d) **and relative height distribution of micelles size of P*n*BuA-*b*-PAA film before sputtering **(a)**, after sputtering **(b)**, after thermal annealing **(c)**.

In order to exclude the first hypothesis, we consider the height of circular domains (obtained by section analysis) versus annealing time. Such a graphic (Figure 3) shows that the height of circular domains remains constant (〈*z*〉 = 1.69 nm) after annealing treatment. From the AFM images the circular domains' height distributions were determined by using a software (Nanoscope IIIa) that defines each circular domains area by the surface image sectioning of a plane that was positioned at half micelle height. Each height distribution of circular domains was calculated on a statistical population of 50 circular domains. Each distribution was then fitted by a Gaussian function (the continuous line in each figure) which peak position was taken as the mean value and which FWHM (full width at half maximum) as the deviation on such mean value. The graphic of Figure 3 shows us that annealing process does not change circular domains' height and this fact allows us to exclude the first hypothesis: the preferential diffusion of gold driven by block copolymer template. On the other hand, the thermodynamic basis of hypotheses 1 and 2 is the surface-free energy minimization of the hybrid gold/polymer system. In fact, generally, cluster growth is regulated by the vapour pressure at the surfaces of the cluster, *P*(*R*), depending on the curvature of the surface and it is driven by the minimization of the total surface free energy. For spherical clusters with a radius *R*, the vapour pressure at the surface of the cluster is given by the following relation according to the Gibbs-Thompson equation [38]

(1) $$P(R)=P_{\infty }\exp(2\gamma \Omega /Rk_{\text{B}}T)\approx P_{\infty }(1+c/R)$$

with *P*_∞ _the vapour pressure at a planar surface, γ the surface free energy of gold, *Ω *the atomic volume of gold, *k*_B _the Boltzmann constant, *c *a temperature-dependent but time-independent constant and depending on the diffusion atomic coefficient *D *of gold. The hypothesis 1 involves a surface diffusion of gold on block copolymer surface characterized by a surface diffusion coefficient *D*_s_. The hypothesis 2 involves, instead, a diffusion of gold into the polymer characterized by a diffusion atomic coefficient *D*_in _of gold in the polymer. Obviously, usually, *D*_s _≫ *D*_in_. Just this purely thermodynamic consideration supports the exclusion of hypothesis 2. Nevertheless, for example Kunz [37] observed an in-depth diffusion of gold in polystyrene after annealing. Therefore, we performed the step-by-step TOF-SIMS imaging in order to exclude experimentally and directly the first and the second hypotheses.

We have investigated all the three different steps: (1) HPLB film, (2) hybrid bilayer AuNPs/BCs, (3) annealed hybrid bilayer.

**Figure 4:**
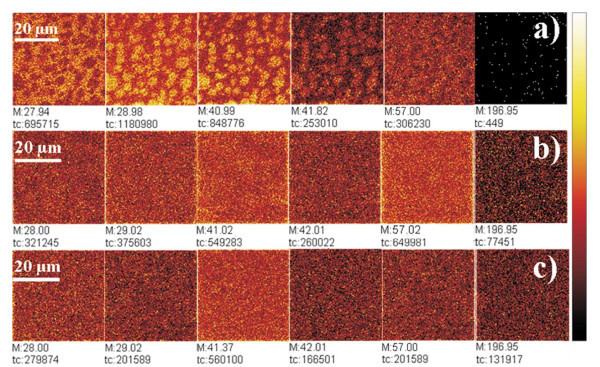
**ToF SIMS chemical maps of each deposition step of as deposited and annealed samples**: **(a) **TOF-SIMS chemical maps of the HP-LB film of P*n*BuA-*b*-PAA; **(b) **TOF-SIMS chemical maps of the HP-LB film of P*n*BuA-*b*-PAA covered with Au nanoparticles deposited by sputtering; **(c) **TOF-SIMS chemical maps of the annealed bilayer (115°C, 15 min) composed by HP-LB film of P*n*BuA-*b*-PAA covered with Au nanoparticles deposited by sputtering.

Figure 4a refers to TOF-SIMS chemical maps of layer obtained at air/water interface and deposited on SiO_2_/Si substrate. Figure 4b refers to TOF-SIMS chemical maps of the annealed bilayer composed by HP-LB film of P*n*BuA-*b*-PAA covered with Au nanoparticles deposited by sputtering. The presence of gold film anneals phase difference of the hybrid bilayers.

In Figure 4a we observe the results of separation phase phenomena and the presence of circular domains in HP-LB film of P*n*BuA-*b*-PAA (Figure 4a). In particular, the bidimensional distributions of the normalized intensities of some molecular fragments (*m*/*z*: 28, 29, 41, 42, 57 and 197 Da that correspond to CO^+^, CHO^+^, C_2_HO^+^, C_2_H_2_O^+^, C_4_H_9_^+ ^and Au^+^, respectively) are shown and the complementarity between PAA molecular fragments (*m*/*z*: 28, 57 Da) and P*n*BuA molecular fragments (*m*/*z*: 29, 41, 42 Da). After gold sputtering deposition we observe the annealing of inhomogeneous composition of the film and the homogeneous surface distribution of gold ion (Figure 4b). Finally, also in Figure 4c the homogeneous distributions of all of the fragments are shown.

In summary, TOF-SIMS imaging allows us to exclude again the first hypothesis, as we know because of the experimental evidence shown in the graphic of Figure 3, but allows us to exclude also the second hypothesis (regarding the in depth diffusion of gold) because we have no evidence by SIMS imaging of gold depletion phenomenon and its diffusion under block copolymer film, in fact we observe an homogeneous distribution of molecular fragments in the uppermost layer after annealing (Figure 4c).

**Figure 5:**
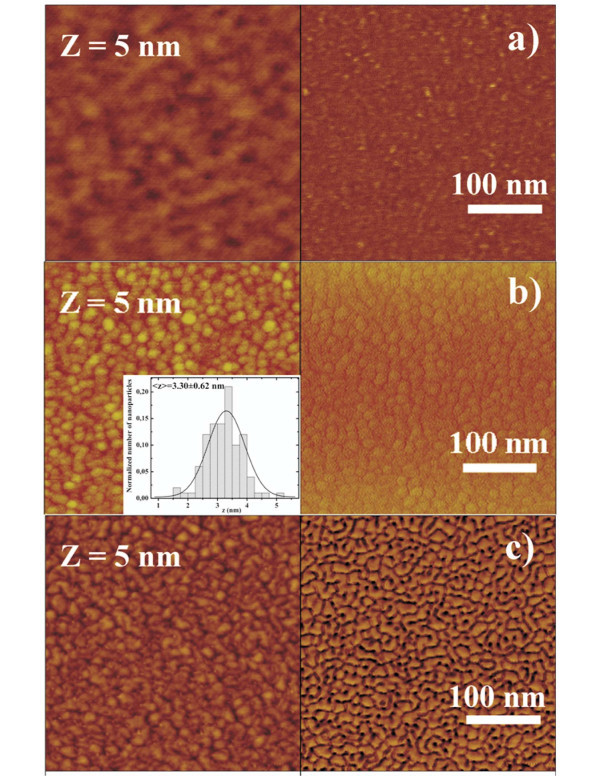
**Nanometric scale AFM images of each deposition step of as deposited and annealed samples**: **(a) **AFM images in detail (around micelles) of P*n*BuA-*b*-PAA film; **(b) **AFM images of gold nanoparticles after sputtering deposition, inset: Gaussian distribution of gold nanoparticles' size (height). **(c) **AFM image in detail of annealed (115°C, 15 min) hybrid bilayer with evidence of the modification of gold film nanostructures.

Gold nanoparticles layer, shown in AFM images of Figure 5, are characterized by a specific value of height (*z *= 3.3 nm) obtained with accurate experimental conditions of sputtering deposition. From the AFM images the Au NPs height distributions were determined by using a software (Nanoscope IIIa) that define each nanocluster area by the surface image sectioning of a plane that was positioned at half cluster height. The height distribution (Figure 5b inset) of the Au NPs was obtained on a statistical population of 100 NPs.

By means of the comparison of the nanometric scale morphology before and after the thermal annealing (Figure 5b,c) we observe the nanostructures modification induced by annealing. The new morphology of gold nanostructures is apparently independent on the morphology at the nanoscale of the block copolymer film as we can deduce by the comparison of Figure 5a and 5c.

In summary, hybrid bilayer exhibits a memory effect induced by thermal annealing and these effects can be explained by third hypothesis that takes into account only a modified surface-tip interaction induced by thermal annealing. Such hypothesis is supported by the comparison of the nanometric scale morphologies of the block copolymer film, of hybrid bilayer and annealed hybrid bilayer shown in the AFM images of Figure 5. In fact, after thermal annealing, above *T*_g _temperatures, of both of the blocks, the uppermost modified nanostructured gold layer (shown in Figure 5c), become sensitive to the immediately underlying block copolymer film, probably due to the increased diffusion of gold onto diblock copolymer film during the annealing (higher mobility of polymer chains). When gold atoms are sputter-deposited at room temperature onto insulator substrates they, generally, grow in the Volmer-Weber mode forming three-dimensional clusters [39,40]. It is a consequence of the fact that the surface free energy of gold (1.5 J/m^2^) is higher than that one of the insulator substrates (typically in the range 10-100 mJ/m^2^). In general, this growth mode for gold on polymers surfaces also occurs (for example the surface energy of P*n*BuA is about 37 mJ/m^2^) [37,41-43]. As a consequence, a low adhesion energy (*E*_d_) for the gold on polymer substrates is obtained (with respect to gold deposited on metallic or semiconductor substrates). Thermal annealing determines a modification of surface morphology of the gold nanostructures and an increase of the adhesion energy of the gold with P*n*BuA block (*E*_d1_) and with pAA block (*E*_d2_). The different values of *E*_d1 _and *E*_d2 _determine the interaction modification of the tip with gold on circular domains (constituted by block 1 PAA) and with gold on the remaining matrix (constituted by P*n*BuA) resulting in the return of two different phases.

## Conclusions

The organization of metallic nanoparticles within polymer films can be achieved using many routes. Our method exploits the self-organization characteristic of sputtered Au nanoparticles on self-assembled P*n*BuA-*b*-PAA film obtained by HP-LB method. We studied the morphology and the phase-separation of the film before and after Au sputtering. The effect of the increased mobility of the polymer chains onto the nanoparticles' organization has been studied by heating the polymer-Au bilayer at *T *>*T*_g_.

The nanoparticles' distribution onto the block copolymer domains, studied by AFM and TOF-SIMS, seems strongly affected by the bilayer annealing. In particular, hybrid bilayers exhibit memory effects as a consequence of thermal annealing. Such effects are proved by morphological and compositional experimental evidence of Au NPS/Block copolymer hybrid bilayer and can be explained by the hypothesis that takes into account a modified surface-tip interaction induced by thermal annealing. Such hypothesis is supported by the comparison of the nanomorphologies of the block copolymer film, of hybrid bilayer and annealed hybrid bilayer shown in the AFM images. In fact, after thermal annealing, above *T*_g _temperatures of both of the blocks, the uppermost modified nanostructured gold layer becomes sensitive to the immediately underlying block copolymer film, probably due to the increased diffusion of gold onto diblock copolymer film during the annealing. In particular, thermal annealing determines a modification of surface morphology of the gold nanostructures and an increase of the adhesion energy of the gold with P*n*BuA block (*E*_d1_) and with PAA block (*E*_d2_). The different values of *E*_d1 _and *E*_d2 _determine the interaction modification of the tip with gold on circular domains (constituted by block 1 PAA) and with gold on the remaining matrix (constituted by P*n*BuA) resulting in the return of two different phases. Furthermore, annealing at *T *>*T*_g _does not induce polymer mixing between two blocks or between blocks and gold.

## Abbreviations

AFM: atomic force microscopy; CMC: critical micellar concentration; HP-LB: horizontal precipitation Langmuir-Blodgett; NP: gold nanoparticle; P*n*BuA-*b*-PAA: poly-*n*-butylacrylate-*block*-polyacrylic acid; TOF-SIMS: time of flight secondary ion mass spectrometry.

## Competing interests

The authors declare that they have no competing interests.

## Authors' contributions

VT: conceived of the study, and participated in its design and coordination; carried out the diblock copolymer film deposition, the ToF SIMS imaging and the atomic force microscopy characterization; interpreted and analyzed the experimental data; drafted the manuscript. FR conceived of the study, and participated in its design; carried out the gold sputter deposition and the annealing processes; participated in the interpretation of the experimental data; contributed in drafting the manuscript. AL participated in the ToF SIMS characterization and in helpful scientific discussion about data interpretation. MGG conceived of the study, and participated in its design; participated in the interpretation of the experimental data; contributed in drafting the manuscript. GM conceived of the study, and participated in its design and coordination; participated in the interpretation of the experimental data; contributed in drafting the manuscript.

All authors read and approved the final manuscript.
